# Maintenance of neutralizing antibodies over ten months in convalescent SARS‐CoV‐2 afflicted patients

**DOI:** 10.1111/tbed.14130

**Published:** 2021-05-27

**Authors:** Sissy Therese Sonnleitner, Martina Prelog, Bianca Jansen, Chantal Rodgarkia‐Dara, Sarah Gietl, Carmen Maria Schönegger, Stephan Koblmüller, Christian Sturmbauer, Wilfried Posch, Giovanni Almanzar, Hanna Jury, Tom Loney, Alexander Tichy, Norbert Nowotny, Gernot Walder

**Affiliations:** ^1^ Department of Virology Medical Laboratory Dr. Gernot Walder GmbH Ausservillgraten Austria; ^2^ Division of Hygiene and Medical Microbiology Medical University of Innsbruck Innsbruck Austria; ^3^ Department of Pediatrics Pediatric Rheumatology/Special Immunology University Hospital Wuerzburg Wuerzburg Germany; ^4^ THP Medical products Vienna Austria; ^5^ Institute of Biology University of Graz Graz Austria; ^6^ Department of Basic Medical Sciences College of Medicine Mohammed Bin Rashid University of Medicine and Health Sciences Dubai United Arab Emirates; ^7^ Department for Biomedical Sciences University of Veterinary Medicine Vienna Vienna Austria; ^8^ Viral Zoonoses, Emerging and Vector‐Borne Infections Group Institute of Virology University of Veterinary Medicine Vienna Vienna Austria

**Keywords:** IgG antibodies, immunity, neutralization test, persistence, SARS‐CoV‐2

## Abstract

Knowledge of the level and duration of protective immunity against SARS‐CoV‐2 after primary infection is of crucial importance for preventive approaches. Currently, there is a lack of evidence on the persistence of specific antibodies. We investigated the generation and maintenance of neutralizing antibodies of convalescent SARS‐CoV‐2‐afflicted patients over a ten‐month period post‐primary infection using an immunofluorescence assay, a commercial chemiluminescent immunoassay and an in‐house enzyme‐linked neutralization assay. We present the successful application of an improved version of the plaque‐reduction neutralization assay which can be analysed optometrically to simplify data interpretation. Based on the results of the enzyme‐linked neutralization assay, neutralizing antibodies were maintained in 77.4% of convalescent individuals without relevant decay over ten months. Furthermore, a positive correlation between severity of infection and antibody titre was observed. In conclusion, SARS‐CoV‐2‐afflicted individuals have been proven to be able to develop and maintain neutralizing antibodies over a period of ten months after primary infection. Findings suggest long‐lasting presumably protective humoral immune responses after wild‐type infection.

## 1 INTRODUCTION

A novel coronavirus emerged around December 2019 in Wuhan (China) and rapidly spread around the world prompting the World Health Organization (WHO) to declare the disease a global pandemic on 11 March 2020. A year after the pandemic began and there is still a lack of data on the persistence of the immunologic footprint left in a convalescent SARS‐CoV‐2 afflicted patient. Moreover, the first publications reported discrepancies concerning the persistence of specific IgG antibodies (Long, Deng, et al., 2020; Ong et al., 2020; Wajnberg et al., 2020). The development of specific antibodies in the acute phase of COVID‐19 is well documented and most patients display specific antibody responses between day 10 and day 21 post‐infection (Kellam & Barclay, 2020; Long, Deng, et al., 2020; Long, Tang, et al., 2020). However, milder courses of disease may result in a delayed generation of antibodies and a small number of patients may even stay antibody negative after infection with SARS‐CoV‐2 (Long, Tang, et al., 2020). In comparable studies, the seropositivity rate reached up to 90% (Nie et al., 2020) and 100% (Zhang et al., 2020) within 20 days post‐infection. Studies investigating the persistence of the antibody response are rare; however, it is known that antibodies to other coronaviruses wane over time from the onset of symptoms to between 12 and 52 weeks (Kellam & Barclay, 2020). Short follow‐up studies have shown preservation of SARS‐CoV‐2 IgG antibody levels over the course of seven weeks (Xiao et al., 2020), in at least 80% of patients (Lu et al., 2020).

A retrospective observational study in Austria proved the persistence of protective antibodies in SARS‐CoV‐2 survivors 6 months post‐infection (Pilz et al., 2021). In comparison, 90% and 50% of SARS‐CoV‐1 infected patients were shown to maintain IgG antibodies for two and three years, respectively (Wu et al., 2007).

Short‐term immunity is defined as the wane of specific antibodies after a period of approximately 40 weeks leading to annual or otherwise periodic outbreaks which are well‐known from non‐SARS‐like human coronaviruses as well as influenza viruses (Kissler et al., 2020). In order to understand the possible future scenarios of herd immunity after wild‐type infection, it is vital to quantify the duration of protective immunity against SARS‐CoV‐2 following primary infection.

In this study, we followed 34 volunteers representing the first SARS‐CoV‐2 afflicted patients in East Tyrol, a geographically separated region in Austria with a population of approximately ~50,000, which was one of the first central European hotspot areas affected by SARS‐CoV‐2 after the outbreak cluster in Ischgl, Tyrol. We investigated the course of specific antibody responses starting 21–43 days after disease onset until 10 months (40 weeks) post‐infection.

To obtain a reliable overview of the development of antibodies over time, we chose to compare three different serologic methods with different diagnostic targets. Specifically, we detected IgG antibodies targeting Spike 1 and 2 via chemiluminescent immunoassay, polyclonal IgG and IgM antibodies via immunofluorescence assay, and neutralizing antibodies using an in‐house enzyme‐linked neutralization assay (ELNA). A technique similiar to ELNA was recently used for SARS‐CoV‐2 serology by different research groups (Amanat et al., 2020; Park et al., 2020). This method is faster than a plaque‐reduction neutralization assay and allows the determination of neutralization antibodies within 30 hr.

## 2 MATERIAL & METHODS

### 2.1 Enrolment and sample collection

Thirty‐four volunteers in East Tyrol with PCR‐confirmed SARS‐CoV‐2 infections and mostly clinically manifested COVID‐19 symptoms (31/34) were followed over 10 months post‐infection. Ethical approval to use residual routinely taken serum samples for retrospective antibody analyses was obtained by the Ethics Committee of the University Hospital Wuerzburg (no. 20201105_01). All participants provided written informed consent and the study was performed according to the principles of the declaration of Helsinki 2013.

Following the initial baseline data collection, we conducted serological follow‐up examinations to evaluate seroconversion rates. First blood draw occurred 21–43 days (mean 33 days, standard deviation 5.7 days) after the onset of symptoms in the first week of April 2020 (T1, one‐month post‐infection), T3 (three months post‐infection), at T5 (five months post‐infection) and a final blood draw at 10 months post‐infection in February 2021, called T10. Clinical data were obtained using a standardized data collection form.

Disease severity for each patient was assessed clinically using a standardized questionnaire including age, gender, pre‐existing as well as acute physical condition (Gietl et al., 2021) and rated as asymptomatic, mild, moderate or severe course of disease according to the definitions previously published (Gietl et al., 2021).

The presence of different types of antibodies was analysed in follow‐up serum samples by three different serologic methods to ensure validity of results: IgG in‐house immunofluorescence assay (IFA), IgM in‐house IFA, CLIA IGG^®^ SARS‐CoV‐2 S1/S2 IgG (DiaSorin S.p.A.) and enzyme‐linked neutralization assay (ELNA).

### 2.2 RNA extraction and RT‐qPCR

Nasopharyngeal swabs were taken by trained healthcare personnel. Immediately after collection, viral RNA was extracted using the Indimag Pathogen kit (Indical Bioscience GmbH) and tested for SARS‐CoV‐2 by RT‐qPCR using the Bio‐Rad CFX96 system (Roche) with a LightMix Modular Assay kit in accordance with the modified Charité guidelines (Corman et al., 2020). 10 µl of extracted RNA was added into 15 µl reaction mixture (mastermix). Each 15 µl mastermix contained 12.5 µl buffer solution, 0.25 µl enzyme mix, 1.75 µl of nuclease‐free water and 0.5 µl primer probe wHCoV (ORF1ab: E‐Gene, occasionally N‐Gene/Rd‐Gene). Reactions were incubated at 55°C for 5 min and 95°C for 5 min for reverse transcription of viral RNA, sample denaturation and enzyme activation. These steps were followed by PCR‐amplification with 45 cycles at 95°C for 5 s, 60°C for 15 s and 72°C for 15 s. Cooling was implemented at 10°C for 30 s.

Results were interpreted based on the second derivative maximum (SDM) method (Tichopad et al., 2003). Positive results were confirmed by RdRp‐ and N‐gene (Corman et al., 2020) RT‐qPCR. A cycle threshold (Ct) value higher than 40 was defined as negative.

### 2.3 Isolation of SARS‐CoV‐2

Isolation of SARS‐CoV‐2 was attempted from RT‐qPCR positive nasopharyngeal swabs by inoculation on VeroB4 (no. ACC‐33, DSMZ) in T25 tissue culture flasks for 1 hr at 35°C. After incubation, the sample was removed and Medium199 (Gibco) with 2.5% foetal calf serum (FCS; Gibco) and a mixture of antibiotics (streptomycin, vancomycin, penicillin, each 1 µg/ml) was added. We monitored virus cultures daily for cytopathic effects and tested for specific viral RNA every three days. Isolation was considered successful when the cytopathic effect was 80%–100% in passage 0 as well as passage 1 and/or Ct value in qPCR was lower than 20. Highly positive supernatants were harvested, centrifuged at 3,400 *g* for 5 min and stored at −80°C in 10% FCS. A further passage of diverse isolates was performed to obtain the highest possible concentration, which was Ct 14 on average. All work involving infectious SARS‐CoV‐2 was carried out in a BSL3 facility, following the institutional guidelines and regulations. Whole‐genome sequencing was carried out by Eurofins Genomics, Germany, and a frequently detected local genotype (GISAID accession number hCoV‐19/Austria/CeMM1012/2020|EPI ISL 583853|2020‐03‐27) was used for the neutralization assay.

In‐house immunofluorescence assays (IFA) were assembled as described elsewhere (Sonnleitner et al., 2014). In brief, VeroB4 cells were infected with the local SARS‐CoV‐2 strain EPI ISL 583853 and fixed on IFA slides after 3 days using ice‐cold acetone‐methanol (1:1).

The LIAISON^®^ SARS‐CoV‐2 S1/S2 IgG (DiaSorin S.p.A.) (LIAISON) is a CLIA (Chemiluminescent Immunoassay) which detects IgG antibodies reactive with the spike protein (S1/S2 domain). The assay was performed on the LIAISON^®^ XL Analyzer according to the manufacturer's instructions. The diagnostic sensitivity was 97.9% (89.1%–99.6%; Wilson 95% CI) according to the manufacturer, the specificity in laboratory routine was 99.0% (96.4%–99.7%; Wilson 95% CI).

### 2.4 Enzyme‐linked neutralization assay (ELNA)

VeroB4 cells (ACC‐33, DSMZ) were seeded in flat‐bottom 96 well plates with Medium199 (Thermo Scientific Gibco) and 10% foetal calf serum (Thermo Scientific Gibco) at a density of about 10^6^ cells/ml to give a confluent monolayer. Next day, an infectivity titration was carried out to determine 100 tissue culture infectious dose 50% (100 TCID_50_) (Vihay, 2019; Ramakrishnan, 2016; Amanat et al., 2020). Sera were heat inactivated by incubation at 56°C for 30 min. All sera were primarily tested via a classical plaque‐reduction neutralization test (PRNT) according to previously published data (Vihay, 2019). To evaluate the cut‐off titre for the PRNT, 100 sera of healthy East Tyrolean blood donors from the pre‐pandemic years 2012 and 2013 were tested in SARS‐CoV‐2 specific PRNT and ELNA. The cut‐off titres were set at 1:32 with a viral solution of 100 TCID_50_ for PRNT and 1:4 with a viral solution of 1 × 10^5^ TCID_50_ for ELNA.

With these evaluated sera, we adapted the PRNT to an ELNA without the need of an apparent cytopathic effect (CPE) and a shorter incubation period of <24 hr. For ELNA, sera were titrated in duplicate in twofold dilution steps, starting at a dilution of 1:4 in Medium199 containing 3% foetal calf serum. Equal volumes of virus (1 × 10^5^ TCID_50_) and serum dilutions in Medium199 were mixed and subsequently incubated for 1 hr at 35°C in U‐bottom 96 well plates (Thermo Scientific Nunc, USA). After incubation, a pre‐seeded flat‐bottom 96 well plate with confluent VeroB4 cells was used, medium was discarded, the incubated mixture of patient's serum and defined virus solution was transferred to each corresponding well of the flat‐bottom plate and the plate was incubated for 24 hr at 35°C. Incubation was stopped by discarding supernatant, cells were washed in PBS twice, fixed with ice‐cold acetone‐methanol (1:1) and frozen for at least 15 min. All steps were performed under strict observation and in compliance with biosafety level 3. The analysis was carried out like an enzyme‐linked immunosorbent assay using a BEP III (Siemens) according to the following steps: blocking (45 min, 37°C, Candor Biosciences), washing 3× (wash pod, Siemens, Germany), anti‐SARS‐CoV‐2 nucleocapsid protein IgG (Bioss bsm‐41413M, dilution 1:5,000 for 30 min. at 37°C), washing 3×, adding of horseradish‐peroxidase‐conjugated goat anti‐mouse IgG (ABIN376241, dilution 1:5,000 for 30 min. at 37°C), washing 3×, adding substrate tetramethylbenzidine (TMB) and stop solution (Siemens). The cut‐off titre was set by titrating defined negative human sera from volunteers out of healthy Tyrolean blood donors from the year 2009 and was set at 1:4 in combination with the viral dose of 1 × 10^5^ TCID_50_ and calculated as median optic density minus the standard deviation. A sample was considered positive when the given optic density was higher than the cut‐off titre.

Patients’ sera were tested in duplicate in ELNA and were scored as follows: titres of 1:4 as weak neutralization; titres of 1:8 or 1:16 as moderate/good and >1:16 as strong neutralizing ability. Sera with single titres between 1:4 and negative were valued as borderline.

### 2.5 Definition of antibody development

In ELNA and IFA, a change in antibody titre of more than one dilution between time point 1 (T1) and time point 10 (T10) was defined as an increase or decrease of the titre. In the IgG CLIA assay, the development of antibodies was defined as a change in chemoluminescence of more than 50%.

### 2.6 Statistics

Dichotomous data were tested by a chi‐squared test or Fisher's exact test in the case of small group size (*n* < 60) (Microsoft^®^ Excel^®^, Microsoft 395 MSO, Windows 2010). A two‐sided significance level of *p* = .05 was used for determining statistical significance. The Spearman's rank correlation coefficient was used to analyse correlation of titres between CLIA and ELNA (Microsoft^®^ Excel^®^, Microsoft 395 MSO, Windows 2010). To determine the predominant titres in a group of patients, the median was calculated (Microsoft® Excel®, Microsoft 395 MSO, Windows 2010) according to McHugh, 2013. After testing for distribution (Kolmogorov–Smirnov test), non‐parametric continuous independent variables were compared using Mann–Whitney U‐test for each time point. Wilcoxon test was used to measure titre changes from T1 to T5, as well as from T1 to T10.

## 3 RESULTS

### 3.1 Characteristics of the study group

Within our study group, three patients (8.8%) were asymptomatically infected with SARS‐CoV‐2, 20 patients (58.8%) showed a mild, six (17.6%) a moderate and five (14.7%) a severe course of the disease.

In total, the group consisted of 18 women and 16 men. There were no gender‐related differences in the course of disease (*p* = .11), although only one (5.6%) female had a severe course compared to four male patients (25.0%).

The mean age was 49.0 years (range 11–77 years; *SD* = 16.4). Due to expected differences in disease course (Gietl et al., 2021), three age groups were formed 11–30 years, 31–60 years and 61–77 years. Characteristics of the study group are shown in Table 1.

**TABLE 1:** Overview over the participant characteristics

Age [years]	Female [%]	Male [%]	Total	Asymp [%]	Mild [%]	Moderate [%]	Severe [%]	Total
11–30	3 [75.0]	1 [25.0]	4 [100.0]	1 [25.0]	2 [50.0]	0 [0.0]	1 [25.0]	4
31–60	11 [50.0]	11 [50.0]	22 [100.0]	2 [9.1]	13 [59.1]	4 [18.2]	3 [13.6]	22
61–77	4 [50.1]	4 [50.0]	8 [100.0]	0 [0.0]	5 [62.5]	2 [25.0]	1 [12.5]	8
Total	18 [52.9]	16 [47.1]	34 [100.0]	3 [8.8]	20 [58.8]	6 [17.6]	5 [14.7]	34

N<del author="Sissy Therese Sonnleitner" command="Delete" timestamp="1620220393544" title="Deleted by Sissy Therese Sonnleitner on 5.5.2021, 15:13:13" class="reU3" id="edit6">ote</del>

Thirty‐four volunteers in East Tyrol with PCR‐confirmed SARS‐CoV‐2 infections and mostly clinically manifested COVID‐19 symptoms (31/34) were followed over 40 weeks post‐infection. Asymp, mild, moderate, severe stands for asymptomatic, mild, moderate, severe course of disease.

### 3.2 Seroconversion assessed by different methods

A summary of the titre developments in the four different serologic methods is given in Table 2.

**TABLE 2:** Comparison of different serologic methods for antibody detection in 34 COVID‐19 patients at T1, T5 and T10

	Positive	[%]	Negative	[%]	Borderline	[%]	n. a.	Total
T1								
ELNA	30	90.9	2	5.0	1	3.0	1	34
CLIA IgG	28	82.4	5	7.9	1	2.9	0	34
IFA IgG	32	94.1	2	4.9	0	0.0	0	34
IFA IgM	11	32.4	23	67.6	0	0	0	34
Total	34	100.0		2.9		0.0		
T5								
ELNA	29	85.3	5	14.7	0	0.0	0	34
CLIA IgG	27	81.8	5	15.2	1	3.0	1	34
IFA IgG	21	65.6	5	15.6	6	18.8	2	34
IFA IgM	0	0.0	34	100.0	0	0.0	0	34
T10								
ELNA	24	77.4	7	22.6	0	0.0	3	34
CLIA IgG	26	83.9	5	16.1	0	0.0	3	34
IFA IgG	10	33.3	14	46.7	6	20.0	4	34

Note

Numbers of serum samples were stratified into positive (titre 1:4 in duplicate and higher titres), negative (titres <1:4) and borderline (equivocal titres of 1:2 or 1:4).

Abbreviations: CLIA, chemiluminescent immunoassay; ELNA, enzyme‐linked neutralization assay; IFA, immunofluorescence assay; n.a., not assessed.

### 3.3 Seroconversion assessed by ELNA

**FIGURE 1:**
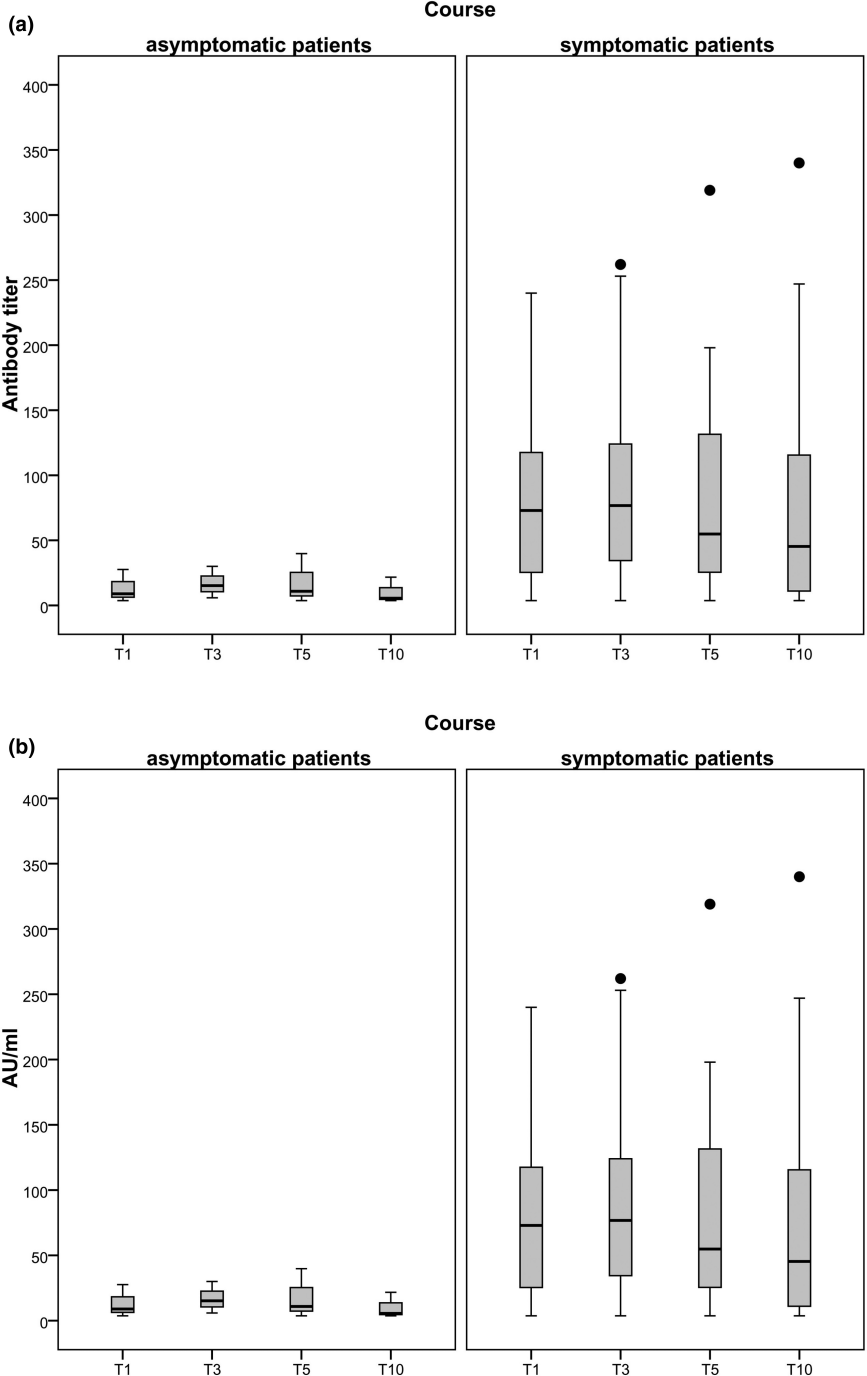
Development of antibody titres (a) and arbitrary units (AU/ml) (b) between patients with an asymptomatic versus a symptomatic course of disease, tested with ELNA (a) and with CLIA S1/S2 IgG (b), respectively. Whiskers are ranging from the 1st quartile (Q1) to the minimum and 3rd quartile (Q3) to the maximum. Outliers are marked as dots when lying more than 1.5× the interquartile range (Q3−A1) above Q3 or as asterisk when lying more than 3× above the Q3. The line inside the box is the 50th percentile (median). Symptomatic patients showed significantly higher AU/ml at T1 and T3 (*p* < .05) (Mann–Whitney *U*‐test)

The majority (90.9%; 30/33) of SARS‐CoV‐2‐positive patients in the convalescent phase (>21 days after symptom onset) tested had seroconverted at T1 in April 2020 and in total, 77.4% of the patients (24/31 samples) had seroconverted until February 2021 (T10), as determined by ELNA (Table 2). Seven patients lost the neutralizing ability against SARS‐CoV‐2 within the study period of ten months whereas one—a male patient with mild symptoms—developed neutralizing antibodies after T3. Overall, more than three‐quarters (77.4%) of SARS‐CoV‐2‐infected persons seroconverted and maintained constant neutralizing antibodies over the study period of 10 months, as shown in Figure 1a.

### 3.4 Seroconversion assessed by CLIA S1/S2 IgG

In CLIA S1/S2 IgG, 83.9% (28/34) of patients seroconverted one month post‐infection and 81.4% (26/31) stayed positive in this test method ten months later without a significant decrease in measurable units of IgG, as shown in Figure 1b.

### 3.5 Seroconversion assessed by IFA IgG

The highest number of positive individuals was found in the IFA IgG one month post‐infection (32/34; 94.1%), which dropped significantly to 65.6% (21/32 tested patients) within the study period of 5 months (*p* = .003) and to 33.3% (10/30 tested patients) after 10 months (*p* < .0001).

### 3.6 Comparison CLIA IgG – ELNA

Both methods showed that specific antibodies stayed constant over the observation period and that greater disease severity led to a more stable antibody response.

CLIA IgG recognized two sera from the first time point as positive (5.9%), which turned out as negative in ELNA. This result is aligned with the manufacturer's reported diagnostic accuracy (i.e., 95% specificity and 100% sensitivity). Repeated serologic investigations at T5 resulted in one patient (3.0%) false negative in CLIA IgG compared to the ELNA method. Ten months post‐infection, the CLIA IgG method recognized 83.9% positive samples (26/31 samples). Two samples that proved positive in CLIA but negative in ELNA had low titres of specific antibodies in CLIA.

### 3.7 Comparison IFA – ELNA

Immunofluorescence assay gave 94.0% positive results at T1 and the strongest decrease of antibodies until T10 with a decrease of positive patients of 60.7% (*n* = 18). Two of the seven sera, which proved negative in IFA, were also negative in ELNA, the others were borderline positive with titres around 1:4 in ELNA, as shown in Table 3.

**TABLE 3:** Development of SARS‐CoV‐2 specific humoral immunity within the first ten months (40 weeks, T10) post‐infection, tested by three different serologic methods

	Decrease	[%]	Increase	[%]	Constant	[%]	Total
NT	16	[51.6]	4	[12.9]	11	[35.5]	31
IFA	22	[71.0]	0	[0.0]	9	[29.0]	31
CLIA IgG	17	[54.8]	12	[38.7]	2	[6.5]	31
IFA IgM	31	[100.0]	0	[0.0]	0	[0.0]	31

Note

Number of serum samples stratified into decreased, increased or constant antibody development over the investigated period of ten months, tested with different serologic methods. Constant variables were defined by non‐significant difference between time point 0 (T0) and time point 10 (T10) by Wilcoxon‐rank test. An increase was defined by a significant increase between T0 and T10. A decrease was defined by a significant decrease between T0 and T10.

Abbreviations: CLIA, chemiluminescent immunoassay; ELNA, enzyme‐linked neutralization assay; IFA, immunofluorescence assay.

The highest number of positive samples was found in the IgG IFA one month post‐infection (32/34; 94.1%), the lowest number of seroconversions was found in CLIA IgG (28/34; 82.4%). In 11 cases (32.4%), IgM was detectable by IFA 28–41 days (T1) after the onset of symptoms.

Four patients (mean age = 51.0 years, *SD* = 12.4) with a mild course of disease did not develop SARS‐CoV‐2 specific antibody responses over the study period and eleven patients developed only weak antibody responses five months post‐infection.

### 3.8 Persistence of IgG antibodies

In one 65‐year‐old male patient with a mild course of disease, the neutralizing antibody titre dropped from 1:32 at T1 to <1:8 at T5 and he lost the specific humoral immune response according to all three serologic methods. Two patients experienced an increase in neutralizing antibodies during the ten‐month follow‐up period. In both patients, the first blood sample showed a weak or moderate neutralizing ability with titres of 1:4 and 1:8, which increased to titres of 1:32 and 1:64, respectively. The course of disease in these cases was mild and severe, respectively. In more than three‐quarters (77.4%) of patients, the neutralizing antibody titres against SARS‐CoV‐2 stayed constant and did not change significantly during the ten‐month follow‐up period. Two patients were negative in the CLIA IgG assay but showed weak neutralizing activities with antibody titres of 1:4.

### 3.9 Influence of disease course on neutralizing antibodies

Symptomatic patients showed significantly higher AU/ml at T1 and T3 (*p* < .05) (Mann–Whitney *U*‐test).

Our data also revealed a relationship between the severity of infection and neutralizing activity by ELNA. Neutralizing titres were compared in a group of 3 asymptomatic versus 31 symptomatic patients, the symptomatic group had higher neutralizing titres approaching significance (*p* = .07).

**FIGURE 2:**
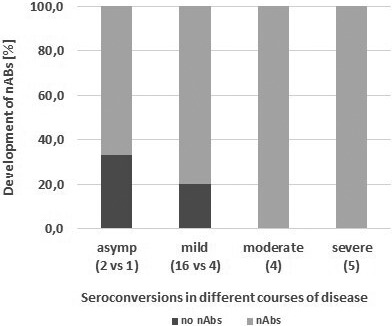
2Percentages of patients with neutralizing antibodies stratified into disease severity. Neutralization activity is more pronounced in patients with more severe infections. Moderate and severe courses of disease led to neutralizing antibodies in 100% of the patients, whereas 33% and 20% of persons with an asymptomatic (asymp) course of infection did not developneutralizing antibodies. Numbers in parenthesis show the number of patients in the particular group. nAbs, Neutralizing antibodies

Moderate or severe SARS‐CoV‐2 infections led to the development of neutralizing antibodies significantly more frequently than in asymptomatic or mild infections (*p* = .03). There was no significant difference in neutralization activities between asymptomatic and mild courses of disease (*p* = .17). Interestingly, 60% of the patients who experienced severe infections (3/5) developed low (titres of 1:4) of neutralizing antibodies (Figure 2).

### 3.10 Age and disease severity

There was no correlation between age and disease severity (*k* = .09) or neutralizing antibodies and disease severeity (*k* = −.0951), nor did we observe a significant predominance of severe cases in oder age groups or male patients (*p* = .53). The mean age of severe cases was 46.0 (*SD* = 15.1, *n* = 5) versus 49.0 years (*SD* = 16.8, *p* = .36, not significant) in cases of other severity categories. All patients with severe infections had neutralizing antibodies at T1. However, only 2 (40%) maintained neutralizing antibodies until T5, and 60% (*n* = 3) in this group showed low titres (<1:8) 5 months post‐infection (T5).

## 4 DISCUSSION

In this study, we evaluated antibody titres in 34 follow‐up sera of SARS‐CoV‐2 afflicted patients one (T1), three (T3), five (T5) and ten months (T10) post‐PCR‐confirmed infection using three different serologic tools ELNA, IFA and CLIA. Our data showed the persistence of specific IgG antibodies during the follow‐up period of ten months and a significant positive correlation between disease severity at initial presentation and neutralization activity. Overall, 77.4% of patients (24/31) maintained specific neutralizing antibodies in ten months post‐infection. More than three‐quarters (78.6%; 22/28) of symptomatic patients and a third (33.3%; 1/3) of asymptomatic patients maintained neutralizing antibodies over the ten‐month observation period. Investigations of IgG levels and neutralizing antibodies in the early phase of convalescence gave a similar result in earlier work (Long, Tang, et al., 2020). More than half (60%; 3/5) of patients with severe infection developed low neutralizing antibody titres (1:4), which suggests that impaired immune responses may contribute to severe disease manifestation; however, detailed clinical and laboratory data regarding the T‐ and B‐cell‐status were missing in our patients. Previous research has shown that B‐cell lymphopenia in severe COVID‐19 afflicted patients was correlated with poor specific humoral immune responses (Melenotte et al., 2020; Ni et al., 2020; Long, Tang, et al., 2020; Wu et al., 2020; Qin et al., 2020). Concomitant treatments (e.g. glucocorticoids or other immunosuppressants recommended for COVID‐19 therapy) may also mitigate the generation of an efficient humoral immune response (Bamoulid et al., 2015; Thaunat et al., 2016).

In accordance with another study (Zhang et al., 2020), the IgG levels in the symptomatic group (*n* = 31) were significantly higher than those in the asymptomatic group (*n* = 3; *p* = .008). Two of these asymptomatic patients were negative in the early stage of the convalescent phase four weeks post‐infection (T1). One patient had a mild course of disease and stayed negative in all tests, whereas another patient with asymptomatic infection developed a weak neutralization response in the late convalescent phase (T5). These findings are aligned with published data, where milder courses of disease may require longer periods to generate specific antibodies and in a low number of cases, patients did not seroconvert at all after infection with SARS‐CoV‐2 (Long, Tang, et al., 2020). Recently published data by Wajnberg et al. (2020) are in line with our results showing relatively stable maintenance of neutralizing antibodies in convalescent COVID‐19 patients over a study period of 5 months. Moreover, another very recent study in Austria concerning re‐infections indicates maintenance of immunity for more than six months post‐infection (Pilz et al., 2021). Specific neutralizing antibodies were detected in 90.0% of laboratory‐confirmed cases of SARS‐CoV‐2 infected patients in the early convalescent phase ~21 days post‐infection and stayed constant in 77.4% of the patients in the convalescent stage ten months later. These observations confirm recently published data on the high prevalence of neutralizing antibodies in most SARS‐CoV‐2 afflicted individuals (Long, Tang, et al., 2020).

In order to perform a valid assessment of serum samples, follow‐up sera were investigated using different serologic methods with differing targets. The ELNA method is a clear improvement of the classical neutralization assay, created from the idea to make the evaluation quantifiable by enzyme‐link whereas the evaluation of a classical plaque‐reduction neutralization test via microscopy or crystal violet staining is reserved for the well‐trained eye of an expert. In the course of the experimental approaches, we discovered and showed the positive side effect that ELNA evaluation does not have to wait for the appearance of a measurable cytopathic effect (CPE). Indeed, ELNA can be carried out 24 hr post‐infection by enzyme‐link using the structural protein N as a target due to its adequate abundance in infected cells, which is an approach that has also been used by others (Amanat et al., 2020). In agreement with previous research (Park et al., 2020), we found that visualizing virus particles and not the CPE leads to identical results in simultaneous test approaches. Furthermore, visualizing virus particles has the advantage of saving time, which is a strong improvement during this stage of the pandemic when immunity and antibody status have an influence on the vaccination recommendations and allocation decisions of vaccine doses in short supply.

The ELNA method is based on the principle that unbound virus particles are able to attach to cells and initiate virus replication which can be determined by staining for SARS‐CoV‐2 (Park et al., 2020), whereas the bound virus particles are washed away by discarding the supernatant after incubation and by the following washing steps. Actually, this method does not directly measure the neutralizing antibodies but indirectly estimates the neutralizing ability in a patient's serum, which can be evoked by different components like specific IgG, IgA or potentially cross‐reactivity with non‐SARS‐like human coronaviruses, suggested by further serological studies in our laboratory (data not shown).

The comparison of different serologic methods shows that the IgG CLIA reflects the results of the neutralization assay with concordances of 95%, giving an overall 6% false‐positive and 3% false‐negative results. Our data show that neutralizing ability is not inevitably correlated with the number of measured units of IgG against S1/S2 and suggests that there could be serological components other than IgG S1/S2 with neutralizing effects; such as IgA, IgM or IgG antibodies targeted against other viral proteins than the spike. However, possibly coating the CLIA assay with spike 1 and 2 in its natural trimeric structure could display better the positions of those important epitopes in vivo. The lack of the trimeric structure of spike 1 and 2 in the setup of the CLIA assay possibly neglects significant binding points. Overall, CLIA data are in agreement with the neutralization assay and the CLIA proves to be a useful tool in laboratories without BSL‐3 and generally for fast antibody testing, as the test can be completed in <1 hr.

The immunofluorescence assay is a method that targets the whole virus, facilitating the visualization of the complete panel of specific antibodies in a patient's serum. In contrast to CLIA, which is coated with spike exclusively, the entire repertoire of epitopes from SARS‐CoV‐2 is presented to the patient's antibodies in the IFA method because the entire virus is presented in and outside the fixed cells.

We interpret the high degree of seroconversion at T1 in IgG IFA (94.1%) as the assay's high binding possibilities to the polyclonal reconvalescent serum and the rapid decline of specific IgG antibodies in IFA with the lower sensitivity of this test system (Cutler and Wright, 1989; Groen et al., 1989; Cunha et al., 2006).

Nevertheless, the IgG IFA was a useful tool in serologic testing at the beginning of the SARS‐CoV‐2 pandemic when no commercial serologic tests were available. The persistence patterns of IgM antibodies in our study follow a typical course in the acute and early reconvalescent phase of a primary infection, with 11% IgM positive patients in T1, all of which seroconverted to IgG in the course of the ten‐month follow‐up period. All three methods show the same pattern of specific antibody levels after infection with SARS‐CoV‐2 and confirm their persistence over the study period of ten months. The ELNA and similar assays represent the gold standard for the neutralizing ability which is why we proclaim the results of this serologic method as most relevant for the question concerning the maintenance of SARS‐CoV‐2 specific humoral immunity.

To conclude, our results show that a high proportion of convalescent SARS‐CoV‐2 afflicted patients maintain constant titres of neutralizing antibodies at least ten months post‐infection, which are expected to be high enough to provide protective and long‐lasting humoral immunity after wild‐type SARS‐CoV‐2 infection. These findings are extremely important to estimate the long‐term efficacy of COVID‐19 vaccinations. Furthermore, our data reveal that individuals who have recovered from symptomatic COVID‐19 generated more robust neutralizing antibody responses than those with asymptomatic infections.

## CONFLICT OF INTEREST

The authors have no conflict of interest to declare.

## ETHICAL APPROVAL

The authors confirm that the ethical policies of the journal, as noted on the journal's author guidelines page, have been adhered to and the appropriate ethical review committee approval has been received (Ethics Committee of the University Hospital Wuerzburg no. 20201105_01).
